# Accumulation of poly(A) RNA in nuclear granules enriched in Sam68 in motor neurons from the SMNΔ7 mouse model of SMA

**DOI:** 10.1038/s41598-018-27821-3

**Published:** 2018-06-25

**Authors:** J. Oriol Narcís, Olga Tapia, Olga Tarabal, Lídia Piedrafita, Jordi Calderó, Maria T. Berciano, Miguel Lafarga

**Affiliations:** 1 Department of Anatomy and Cell Biology and “Centro de Investigación Biomédica en Red sobre Enfermedades Neurodegenerativas (CIBERNED)”, University of Cantabria-IDIVAL, Santander, Spain; 2 Department of Experimental Medicine, School of Medicine, University of Lleida and “Institut de Recerca Biomèdica de Lleida” (IRBLLEIDA), Lleida, Spain; 3 Present Address: Department of Molecular Biology and CIBERNED, University of Cantabria-IDIVAL, Santander, Spain

## Abstract

Spinal muscular atrophy (SMA) is a severe motor neuron (MN) disease caused by the deletion or mutation of the *survival motor neuron 1* (*SMN1*) gene, which results in reduced levels of the SMN protein and the selective degeneration of lower MNs. The best-known function of SMN is the biogenesis of spliceosomal snRNPs, the major components of the pre-mRNA splicing machinery. Therefore, SMN deficiency in SMA leads to widespread splicing abnormalities. We used the SMN∆7 mouse model of SMA to investigate the cellular reorganization of polyadenylated mRNAs associated with the splicing dysfunction in MNs. We demonstrate that SMN deficiency induced the abnormal nuclear accumulation in euchromatin domains of poly(A) RNA granules (PARGs) enriched in the splicing regulator Sam68. However, these granules lacked other RNA-binding proteins, such as TDP43, PABPN1, hnRNPA12B, REF and Y14, which are essential for mRNA processing and nuclear export. These effects were accompanied by changes in the alternative splicing of the Sam68-dependent *Bcl-x* and *Nrnx1* genes, as well as changes in the relative accumulation of the intron-containing *Chat*, *Chodl, Myh9* and *Myh1*4 mRNAs, which are all important for MN functions. PARG-containing MNs were observed at presymptomatic SMA stage, increasing their number during the symptomatic stage. Moreover, the massive accumulations of poly(A) RNA granules in MNs was accompanied by the cytoplasmic depletion of polyadenylated mRNAs for their translation. We suggest that the SMN-dependent abnormal accumulation of polyadenylated mRNAs and Sam68 in PARGs reflects a severe dysfunction of both mRNA processing and translation, which could contribute to SMA pathogenesis.

## Introduction

The processing of mRNAs includes three essential modifications, capping, splicing and polyadenylation, which mainly occur at the sites of transcription (co-transcriptional)^1,2^. An analysis of the human transcriptome revealed that >90% of protein-coding genes undergo alternative splicing, which generates multiple mRNA variants that encode the vast proteomic repertoire required for the protein interactome^3^. Another fundamental modification of most mRNAs is the addition of a polyadenosine tail at the 3′ end. Polyadenylation is a modification necessary not only for the stability of mRNAs but also for their nuclear export^4^. Poly(A)-binding protein nuclear 1 (PABPN1) plays a critical role in polyadenylation by strongly enhancing the processivity of poly(A) polymerase^4^. Upon the completion of co-transcriptional processing, mRNAs are exported to the cytoplasm as parts of messenger ribonucleoprotein particles for translation and degradation. During their nuclear journey, mRNAs associate with several RNA-binding proteins that contribute to the regulation of their processing and exportation.

On their nuclear route, some normal and incompletely spliced mRNAs are trafficked and may be retained in nuclear speckles^2,5–7^. These nuclear compartments store, assemble and recycle splicing factors and are also enriched in poly(A) RNAs and several mRNA processing factors, including PABPN1^8–11^.

Defects in pre-mRNA splicing or polyadenylation often prevent the recruitment of mRNA export factors, resulting in the nuclear retention of immature or aberrant pre-mRNAs^12^. Thus, some abnormal mRNA transcripts may accumulate in nuclear inclusions under pathological conditions, such as oculopharyngeal muscular dystrophy, myotonic dystrophy type 1 and fragile X-associated tremor/ataxia syndrome^6,13–15^. Moreover, we have previously reported that the dysfunction of nuclear RNA processing in the sensory ganglion neurons upon proteasome inhibition induces the nuclear aggregation of polyadenylated mRNAs and the RNA-binding protein Sam68 (src-associated protein in mitosis of 68kD) into a new nuclear structure called the “poly(A) RNA granule” (PARG)^16,17^. The sequestration of crucial RNA-binding proteins in nuclear inclusions or granules may prevent their normal function, and contribute to disease pathogenesis^18^.

Spinal muscular atrophy (SMA) is an autosomal recessive disease characterized by the progressive degeneration and loss of spinal cord and brainstem motor neurons (MNs)^19,20^. SMA is caused by a homozygous deletion or mutation in the *survival of motor neuron 1* (*SMN1*) gene that results in decreased levels of the full-length SMN protein^21,22^. SMA patients carry a nearly identical *SMN1* gene paralogue named *SMN2*, which differs from *SMN1* by a C to T transition in exon 7^22,23^. Although both the *SMN1* and *SMN2* genes encode the SMN protein, approximately 90% of the *SMN2* mRNA transcripts generate an alternatively spliced isoform that lacks exon 7 and encodes a truncated form of the SMN protein (SMN∆7) that is rapidly degraded^24^. Therefore, *SMN2* expression cannot fully compensate for the deficiency of the full-length SMN protein. The best-known function of SMN is the biogenesis of spliceosomal snRNPs (for a review, see^25,26^). Linked to this function, SMN deficiency in SMA produces alterations in this snRNP repertoire and causes widespread splicing defects that result in a severe dysregulation of mRNA metabolism in MNs^27–31^. Splicing alterations in the spinal cord in murine SMA models include widespread intron retention, particularly of minor U12 introns, as well as a time-dependent differential expression of a number of exons associated with neurodevelopmental and cell stress pathways^27–29,31,32^. In addition to the dysfunction of pre-mRNA processing, as a pathogenic factor in SMA, recent studies identified ribosome biology and translation efficiency as key processes affected by SMN depletion, which may also contribute to MN degeneration and SMA pathogenesis^33,34^.

The aim of this work is to analyze the subcellular reorganization of polyadenylated mRNAs and certain RNA-binding proteins induced by the SMN-dependent dysfunction of pre-mRNA splicing in MNs from the SMN∆7 mouse model of SMA. Our results demonstrate the nuclear accumulation of poly(A) RNAs in PARGs enriched in the RNA-binding protein Sam68, which is an alternative splicing regulator of pre-mRNAs^35–37^. The formation of PARGs associates with variations in the alternative splicing regulation of several mRNAs, including the Sam68-dependent *Bcl-x* and *Nrxn-1* mRNAs. Moreover, the massive accumulation of PARGs in MNs is accompanied by cytoplasmic depletion of polyadenylated mRNAs, supporting the existence of a severe dysfunction of both mRNA processing and translation, which may be an important pathogenic factor in SMA.

## Results

### Distribution of polyadenylated RNAs in wild-type MN perikarya

With the exception of histone mRNAs, all mRNAs are polyadenylated and have a poly(A) tail that is essential for their exportation and stability^1,4^. Therefore, *in situ* hybridization of poly(A) RNAs with an oligonucleotide poly(dT) probe, which recognizes the poly(A) tail, is a good approach for studying the perikaryal distribution of global mRNAs in MNs.

**Figure 1:**
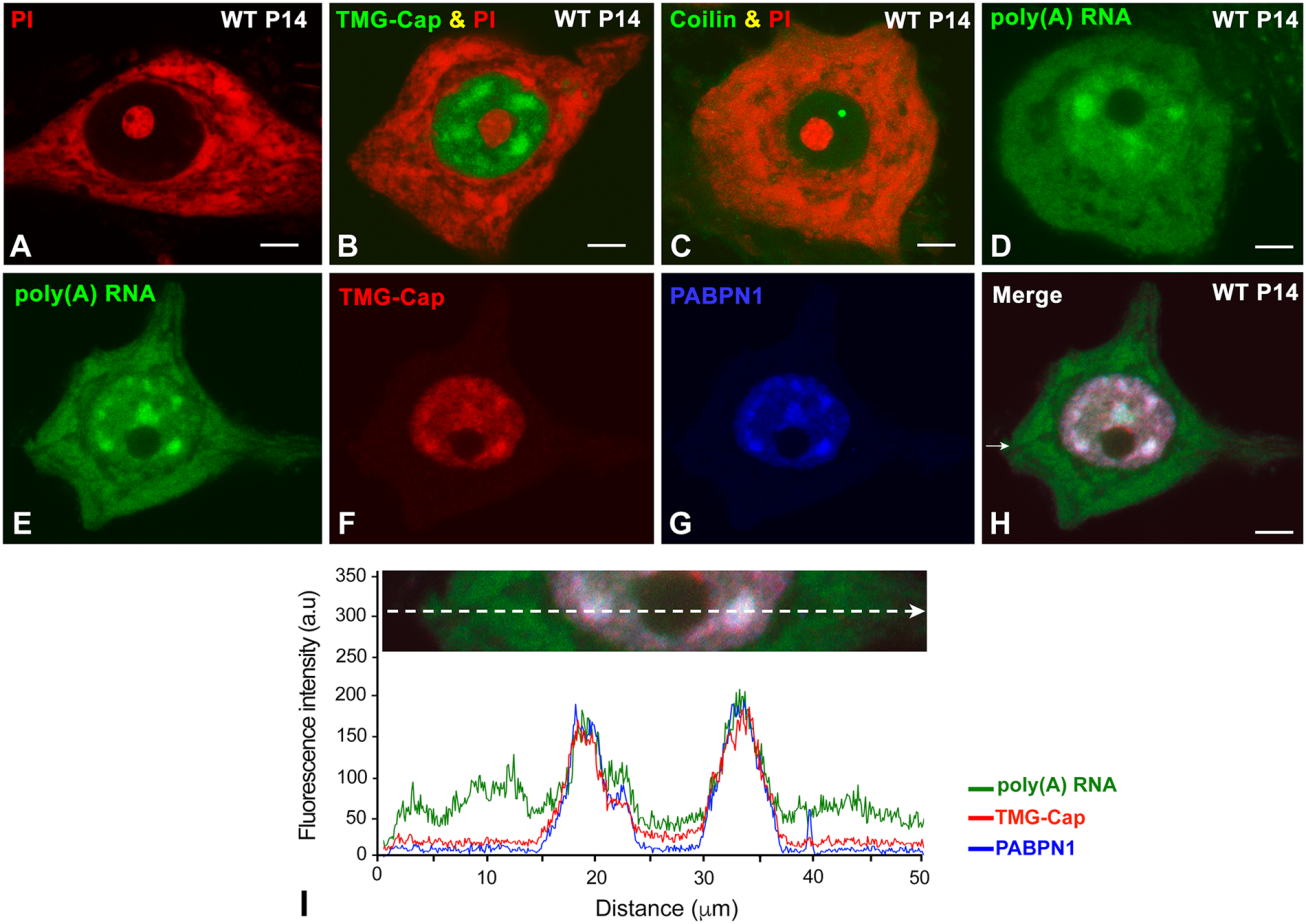
Dissociated spinal cord MNs from WT mice at P14. (**A**) Propidium iodide (PI) staining of the nucleolus and Nissl substance. (**B**) Immunostaining for snRNP splicing factors with the anti-TMG-cap antibody shows the typical concentration of splicing factors in nuclear speckles. The samples were counterstained with PI. **(C**) Immunostaining for coilin illustrates a typical Cajal body free in the nucleoplasm. The samples were counterstained with PI. (**D**) FISH for poly(A) RNAs shows a diffuse nuclear pattern, which excludes the negative nucleolus, in addition to be concentrated in nuclear speckles. In the cytoplasm, poly(A) RNAs accumulate in irregular areas corresponding to the Nissl substance. (**E**–**I**) Triple labeling for poly(A) RNAs, the TMG-cap and PABPN1, demonstrating the colocalization of these three fluorescent signals in nuclear speckles, as revealed by the plot of fluorescence intensity profiles across a line. Scale bar: 5 µm.

After staining with propidium iodide (PI), a cytochemical fluorescent staining for nucleic acid detection, dissociated MNs from wild-type (WT) mice exhibited a prominent nucleolus and large RNA-rich cytoplasmic areas of the Nissl substance, a classic name used to designate the neuronal protein synthesis machinery (for a review, see^38^ (Fig. 1A). Immunolabeling for the TMG-cap and coilin, which are markers of nuclear speckles and Cajal bodies, respectively, revealed the organization of these two nuclear compartments involved in mRNA processing. As shown in Fig. 1B,C, several nuclear speckles and at least one Cajal body were prominent nuclear structures in WT MNs. We next performed fluorescence *in situ* hybridization for the detection of poly(A) RNAs in dissociated WT MNs. Poly(A) RNAs, in addition to being diffusely distributed throughout the nucleus, excluding the nucleolus, were concentrated in nuclear speckles (Fig. 1D,E). As expected, in the cytoplasm, poly(A) RNAs were accumulated in the Nissl substance, the main site of mRNA translation (Fig. 1D,E). Triple labeling for poly(A) RNA in combination with the TMG-cap and PABPN1, two molecular markers of nuclear speckles, demonstrated the accumulation of poly(A) RNAs in these nuclear compartments (Fig. 1E–H), as confirmed by plotting the fluorescence intensity profile across a line (Fig. 1I).

### Polyadenylated mRNA relocalizes in nuclear poly(A) RNA granules in SMN∆7 MNs

**Figure 2:**
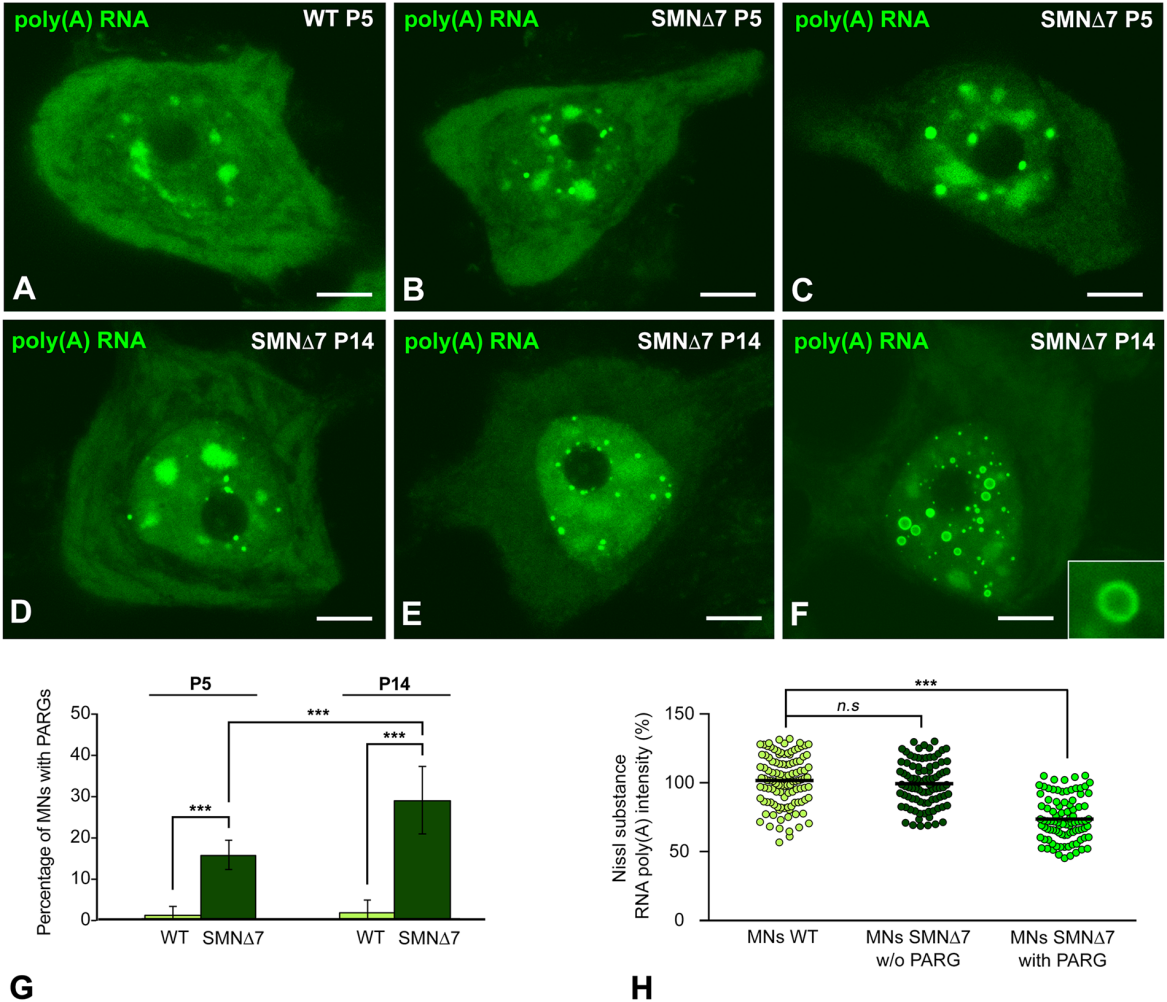
FISH for poly(A) RNAs in WT and SMN∆7 MNs at P5 and P14. (**A**) Typical distribution of Poly(A) RNA concentrated in nuclear speckles and the Nissl substance and diffuse in the nucleoplasm in a WT MN at P5. (**B**,**C**) Formation of PARGs in SMN∆7 MNs at P5. Note the association of PARGs with the nucleolus and nuclear speckles (**B**,**C**) and the reduced hybridization signal in the Nissl substance in panel C. (**D**–**F**) Distribution of poly(A) RNAs in SMN∆7 MNs at P14 showing three progressive stages of PARG accumulation in neuronal perikarya. Note the presence of two categories of PARGs, compact (**D**,**E**) and ring-shaped (**F** and inset), and the cytoplasmic depletion of poly(A) RNAs in MNs containing a large number of PARGs (**E**,**F**). Scale bar: 4 µm. (**G**) Proportion of MNs containing PARGs at P5 and P14. PARGs are already visible at P5 but the percentage of MNs containing PARGs notably increases during the late symptomatic stages (P14); *p* values from data comparison of the percentage of PARG-containing MNs: WT *vs*. SMN∆7 = 3.1E-9 at P5 and 2.7E-8 at P14; SMN∆7 P5 *vs*. SMNΔ7 P14 = 1.5E-4 (***p < 0.0005). (**H**) Scatter dot plots of the fluorescence intensity signals of poly(A) RNAs in the peripheral cytoplasm (Nissl substance) of WT and SMN∆7 MNs at P14. Each dot represents the fluorescence signal of poly(A) RNA intensity from a single MN (mean of four measurements). The horizontal black line represents the mean for each group normalized to 100% of the WT group. A significant reduction in the relative concentration of poly(A) RNAs is observed in PARG-containing SMN∆7 MNs (n = 93) compared with both WT and SMN∆7 MNs without PARGs (n = 112 and n = 90, respectively). *p* = 4.8E-24 from WT and SMN∆7 data comparison (***p < 0.0005).

Next, we analyzed whether the dysfunction of RNA metabolism in SMN∆7 MNs affects the nuclear and cytoplasmic organization of poly(A) RNAs. *In situ* hybridization revealed an important nuclear reorganization of poly(A) RNA in some SMN∆7 MNs during both the late-presymptomatic (postnatal day [P]5) and symptomatic (P14) stages, compared with the staining pattern of WT MNs (Figs 1E, 2A–F). There were notable variations in poly(A) RNA signal intensity in nuclear speckles and, very remarkably, the *de novo* formation and accumulation of round and sharply defined bodies enriched in poly(A) RNA, which were identified as PARGs^16^ (Fig. 2B–F).

The PARGs, which ranged from 0.1 to 1.5 µm in diameter, were distributed throughout the nucleus, excluding the nucleolus. They frequently appeared in close proximity to the nucleolus and nuclear speckles (Fig. 2B,C,E). Two structural configurations of PARGs were found: compact nuclear bodies and ring-shaped structures with a poly(A) RNA-rich ring enclosing a hybridization signal-poor central region (Fig. 2C,F). Quantitative analysis showed a significant increase in the percentage of SMN∆7 MN-containing PARGs at P14 in comparison to the P5 (28% vs 17%) (Fig. 2G). Most commonly, the PARG number ranges from a very few to more than 50 per nucleus. However, some MNs showed very small, countless, PARGs (Fig. 2F).

Interestingly, MNs harboring a large number of PARGs commonly exhibited a weaker poly(A) RNA hybridization signal in the cytoplasm (Fig. 2E,F). Moreover, the cytoplasmic accumulation of poly(A) RNAs in stress granules^39,40^ was not observed in SMN∆7 MNs. Changes in the relative concentration of poly(A) RNA in the cytoplasm were validated by a densitometric analysis of the fluorescence hybridization signal intensities at P14. Several measurements of the poly(A) RNA signal intensities were performed in the peripheral cytoplasm, where the Nissl substance is largely distributed in MNs. A significant reduction of the relative poly(A) RNA concentration was detected in the Nissl substance of PARG-containing MNs, compared with PARG-free MNs, from both SMN∆7 and WT mice (Fig. 2H).

**Figure 3:**
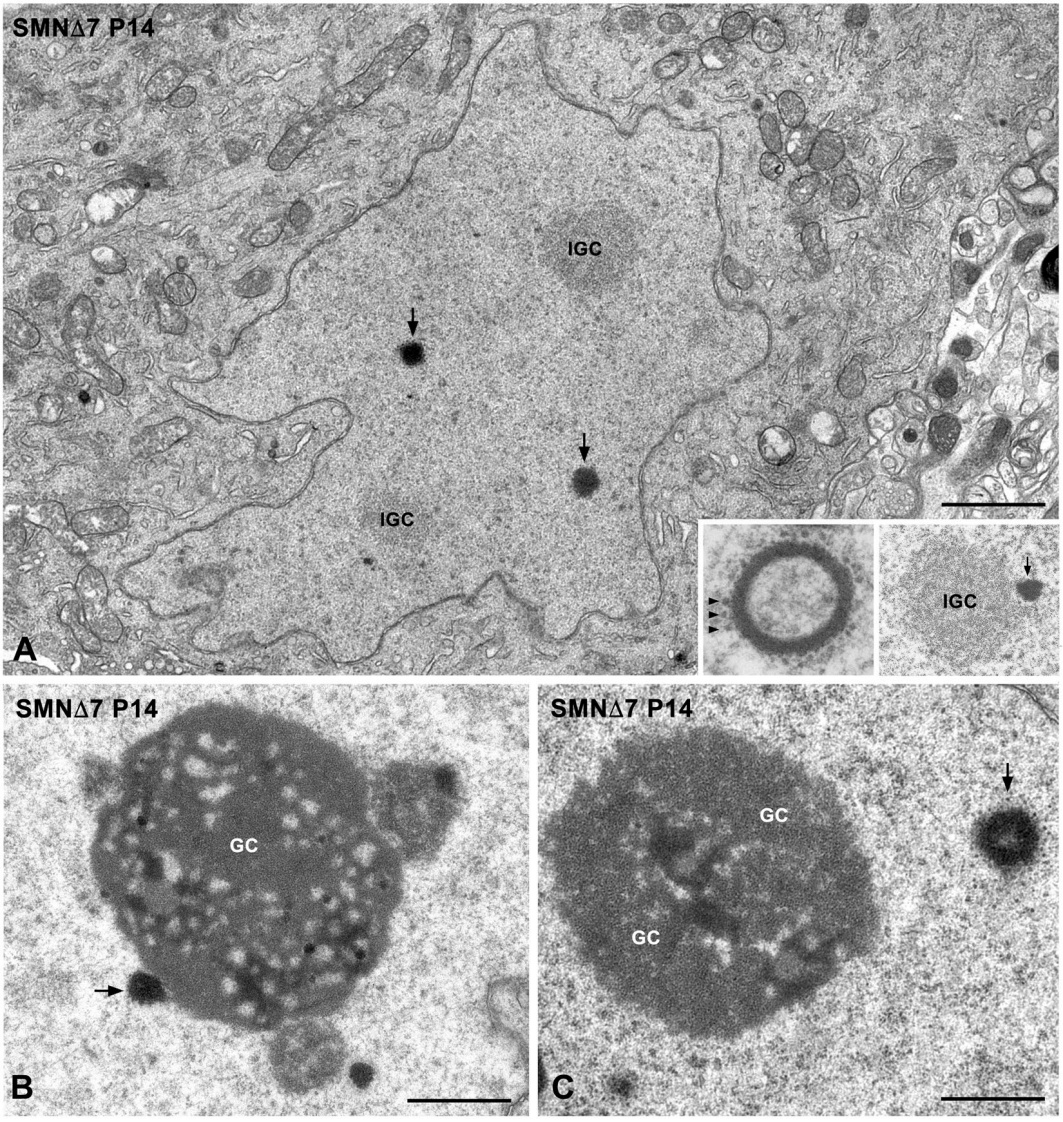
Electron micrograph of a PARG-containing MN during the end-stage of disease (P14). (**A**) The cytoplasm is poor in rough endoplasmic reticulum cisterns; note the abnormal nuclear morphology with numerous invaginations of the nuclear envelope. The nucleus exhibits two compact PARGs (arrows) and two interchromatin granule clusters (IGC). Left inset: detail of a ring-shaped PARGs surrounded by perichromatin granules (arrows). Right inset: interchromatin granule cluster with an associated PARG (arrow). Scale bar: 2 µm. (**B**,**C**) Nucleoli of SMN∆7 MNs at P14, showing the segregation of large masses of the granular component (GC), the presence of a nucleolus-attached PARG (arrow in panel B) and a ring-shaped PARG free in the nucleoplasm and surrounded by perichromatin granules (arrow in panel C). Scale bar: 1 µm.

Electron microscopy analysis of PARG-containing MNs revealed structural features of neuronal dysfunction, including a paucity of the protein synthesis machinery, nuclear shape aberrations with nuclear envelope invaginations (Fig. 3A) and nucleolar segregation of the granular component (Fig. 3B,C), as we have recently reported^34^. Ultrastructural analysis confirmed the presence of two categories of PARGs: rounded, compact electron-dense bodies (Fig. 3A) and larger spherical bodies composed of an electron-dense capsule enclosing an amorphous matrix of very low electron density (Fig. 3A, left inset). In addition, PARGs normally appeared to be closely surrounded by a layer of perichromatin granules (Fig. 3C, left inset), which are sites of storage for hnRNP complexes^41^. Importantly, PARGs localized in euchromatin domains, wherein co-transcriptional pre-mRNA processing occurs^1,2^, and they frequently appeared in close proximity to the nucleolus (Fig. 3B) and interchromatin granule clusters (Fig. 3A, right inset), the ultrastructural counterpart of nuclear speckles^9^.

### The PARG is a distinct nuclear compartment

**Figure 4:**
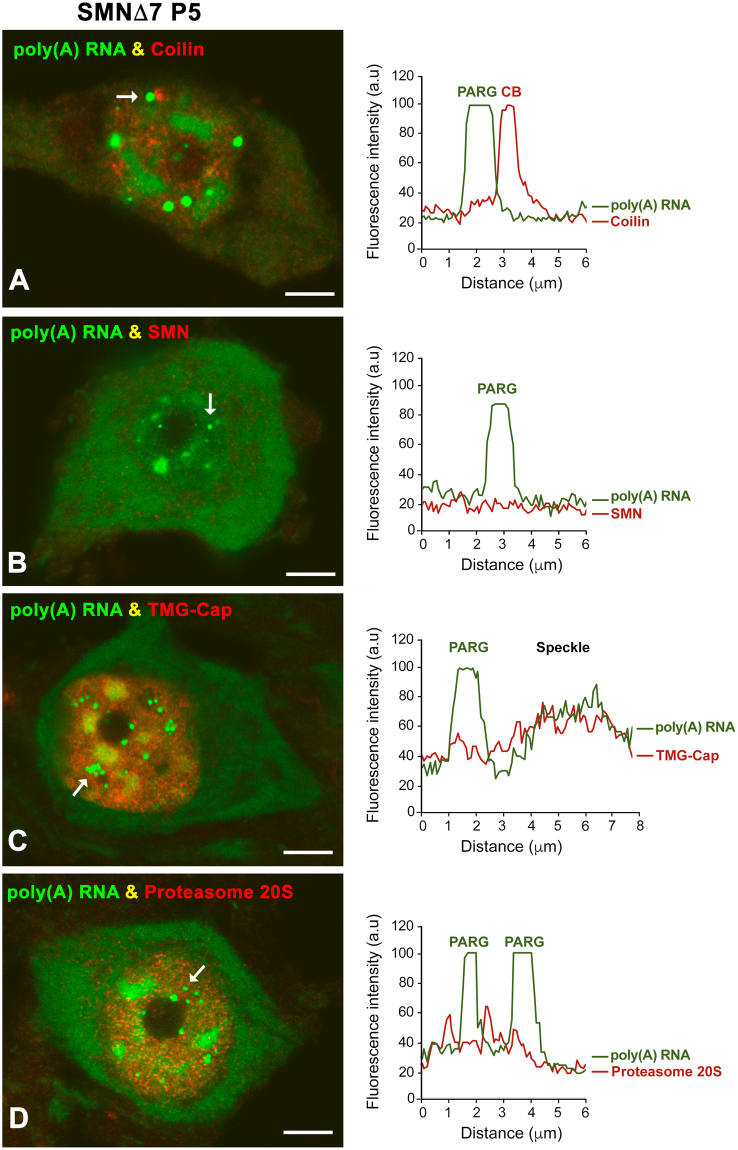
(**A**–**D**) Double labeling for poly(A) RNAs in combination with molecular markers of Cajal bodies (coilin, **A**), nuclear speckles of splicing factors (TMG-cap, **C**), gems (SMN, **B**) and clastosomes (20S proteasome, **D**) demonstrates that the PARG is a distinct nuclear structure. The plot of fluorescence intensity profiles across a line clearly demonstrates a lack of concentration of these molecular markers in PARGs. Scale bar: 4 µm.

To establish the identity of the PARG as a distinct nuclear structure in SMA MNs, we performed double labeling for poly(A) RNA in combination with molecular markers of nuclear compartments, such as coilin (Cajal bodies), SMN (gems), the TMG-cap (nuclear speckles) and the proteasome 20S (clastosomes). In a recent study, we demonstrated that the reduced levels of SMN in MNs from SMN∆7 mice were associated with a severe depletion of Cajal bodies^34^, nuclear structures involved in the biogenesis of both spliceosomal snRNPs and nucleolar snoRNPs (for a review, see^42,43^. Co-staining for poly(A) RNA and coilin confirmed the depletion of typical Cajal bodies in SMN∆7 MNs. Nevertheless, small coilin-positive and poly(A) RNA-negative residual Cajal bodies were occasionally found adjacent to PARGs, but as two clearly distinct nuclear structures (Fig. 4A). Similarly, gems, SMN-positive and coilin-negative nuclear bodies^43,44^, were never observed in SMN∆7 MNs, and SMN was not concentrated in PARGs (Fig. 4B). Moreover, PARGs did not concentrate spliceosomal snRNPs, which typically appeared to be enriched in nuclear speckles immunolabeled for the TMG-cap of spliceosomal snRNAs (Fig. 4C). Finally, the catalytic 20S proteasome, a molecular marker of clastosomes, which are nuclear bodies enriched in ubiquitylated proteins and active 20S proteasomes^45^, was not concentrated in PARGs (Fig. 4D).

### PARGs concentrate the RNA-binding protein Sam68

**Figure 5:**
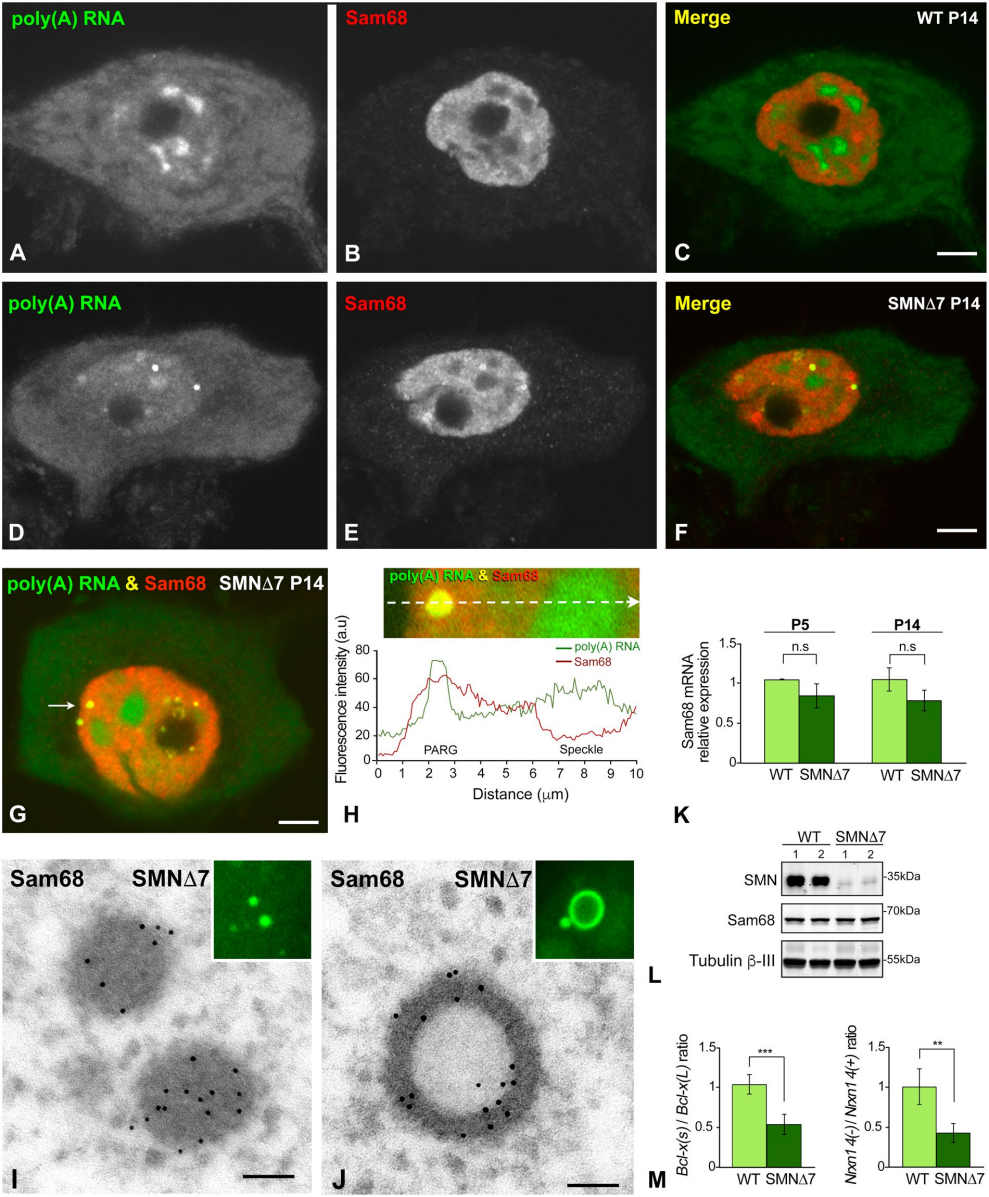
(**A**–**H**) Representative examples of double labeling for poly(A) RNAs and Sam68 in WT (**A**–**C**) and SMN∆7 (**D**–**H**) MNs at P14. (**A**–**C**) In the WT MNs, Sam68 exhibits a diffuse nuclear localization with a few areas of higher intensity. (**D**–**F**) In the SMN∆7 MN, Sam68, in addition to being diffusely distributed throughout the nucleus, appears highly concentrated in two PARGs (**F**). Note the absence of Sam68 in nuclear speckles and the cytoplasmic depletion of poly(A) RNA. Scale bar: 3 µm. (**G**,**H**) The plot of the fluorescence intensity profiles of poly(A) RNAs and Sam68 across a line confirms the colocalization of both signals in a PARG and the concentration of poly(A) RNA, but not of Sam68, in a nuclear speckle. Scale bar: 3 µm. (**I**,**J**) Representative electron micrographs of immunogold electron microscopy localization of Sam68 in dense and ring-shaped PARGs. Scale bar: 200 nm. Insets, FISH detection of poly(A) RNAs in PARGs. (**K**) qRT-PCR of the relative levels of Sam68 mRNA in spinal cord extracts from WT (n = 3) and SMN∆7 mice (n = 5). No significant differences (*n.s*) were found when comparing WT and SMN∆7 samples during both the P5 and P14. (**L**) Representative western blot of Sam68 protein levels showing the dramatic reduction of SMN protein levels in spinal cord lysates from SMN∆7 mice, compared with WT mice, and the absence of significant changes in Sam68 protein levels between WT and SMN∆7 mice at the P14. (**M**) qRT-PCR of the *Bcl-x(s)/Bcl-x(L)* and *Nrxn14*(*−*)*/Nrxn1 4*(+) mRNA ratios in spinal cord extracts from WT and SMN∆7 mice at P14. A significant increase in the relative abundance of the *Bcl-x(L)* and *Nrxn 4(*+) splicing variants was detected in the SMN∆7 mice. *p* values from WT and SMN∆7 data comparison: 3.3E-4 for *Bcl-x(s)/Bcl-x(L)* and 4.4E-3 for *Nrxn1 4*(*−*)*/Nrxn1 4*(+) mRNA ratios (**p < 0.005; ***p < 0.0005).

Since the RNA-binding protein Sam68 is a regulator of *SMN2* alternative splicing^46^, we investigated its nuclear reorganization in SMN-deficient MNs from the SMN∆7 mice. In WT MNs, co-staining for Sam68 and poly(A) RNA revealed a predominant nuclear localization of Sam68, which excluded the nucleolus and poly(A) RNA-positive nuclear speckles (Fig. 5A–C). The nuclear distribution was non-homogeneous, with extensive areas of diffuse staining, and a few irregular domains in which higher levels of Sam68 accumulation were observed (Fig. 5B). Although the basic nuclear pattern of Sam68 immunostaining was preserved in SMN∆7 MNs, this splicing regulator was strongly concentrated in PARGs (Fig. 5D–F). Plot of fluorescence intensity profiles of poly(A) RNA and Sam68 across a line confirmed the colocalization of both signals in PARGs from SMN∆7 MNs, as well as the absence of Sam68 in nuclear speckles (Fig. 5G,H). Similarly, immunogold electron microscopy for Sam68 showed that both the compact and the ring-shaped PARGs were decorated with numerous gold particles (Fig. 5I,J).

We next investigated whether the accumulation of Sam68 in PARGs was associated with changes in Sam68 mRNA and protein levels in tissue extracts from the spinal cord at P5 and P14. Although we observed a trend indicating the downregulation of Sam68 mRNA levels in SMN∆7 mice, this decrease was not significant in the qRT-PCR validation (Fig. 5K). Similarly, western blotting analysis revealed no significant changes in Sam68 protein levels in samples from SMN∆7 mice compared with those from WT littermates (Fig. 5L). However, as expected, a severe reduction of the SMN protein levels was observed in SMN∆7 mice (Fig. 5L).

Previous studies have demonstrated that Sam68 regulates the alternative splicing of two important genes for neuronal function, *Bcl-x* and *Nrxn1*, which encode an apoptotic regulatory factor and the presynaptic membrane protein neurexin, respectively^37,47^. These studies also suggest that the relocation of Sam68 in nuclear foci affects the alternative splicing of its pre-mRNA targets^47^. This finding prompted us to investigate whether the partial relocation of this splicing regulator in PARGs is associated with changes in the balance of *Bcl-x* and *Nrxn1* splicing isoforms. The *Bcl-x* transcript is alternatively spliced to generate the antiapoptotic *Bcl-x(L)* or the proapoptotic *Bcl-x(s)* variants^48^. Real-time PCR quantification of these two variants in spinal cord extracts showed a significant decrease of the *Bcl-x(s)/Bcl-x(L)* ratio in SMN∆7 mice compared with the WT mice (Fig. 5M). Similarly, we found a significant reduction of the *Nrxn1 4*(−)*/Nrxn1 4*(+) ratio in SMN∆7 mice (Fig. 5M), which reflects a higher relative abundance of the isoform *4*(+), which includes exon 20 in the *Nrxn1* alternatively spliced segment 4 (AS4).

### PARGs did not concentrate other RNA-binding proteins involved in mRNA processing and export

Having demonstrated the concentration of Sam68 in PARGs, we then proceeded to investigate the possible accumulation in these granules of other RNA-binding proteins involved in nuclear mRNA processing and export, such as PABPN1, TDP43 (TAR DNA-binding protein 43), hnRNP (heterogeneous nuclear ribonucleoprotein) A2/B1, hnRNP M3, Y14 and REF/Aly.

**Figure 6:**
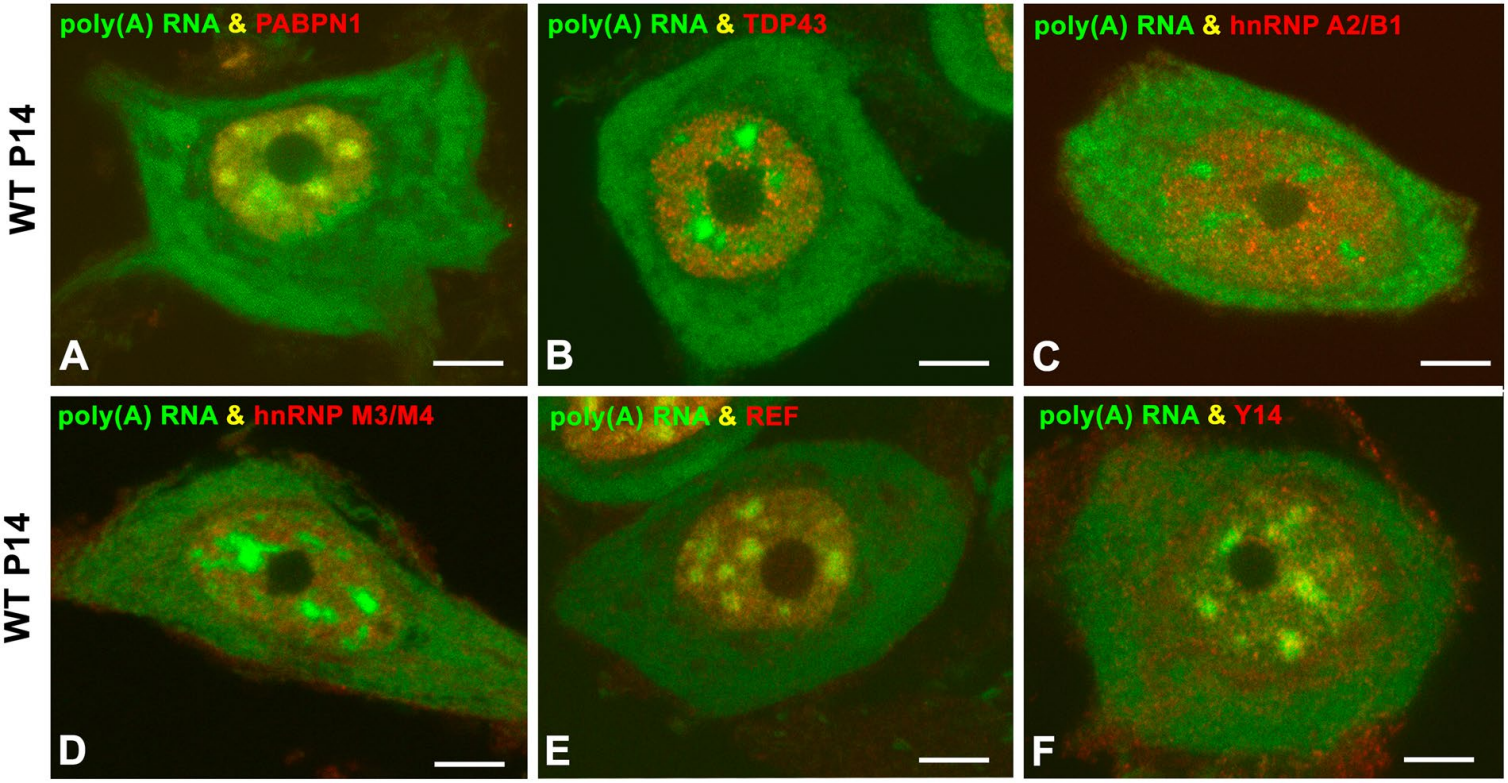
(**A**–**F**) Double labeling for poly(A) RNAs and the RNA-binding proteins PABPN1 (**A**), TDP43 (**B**), hnRNP A2/B1 (**C**), hnRNP M3/M4 (**D**), REF (**E**) and Y14 (**F**) in WT MNs at the P14 stage. Whereas all proteins exhibit a nuclear localization, PABPN1 and REF appear concentrated in nuclear speckles. Scale bar: 4 µm.

**Figure 7:**
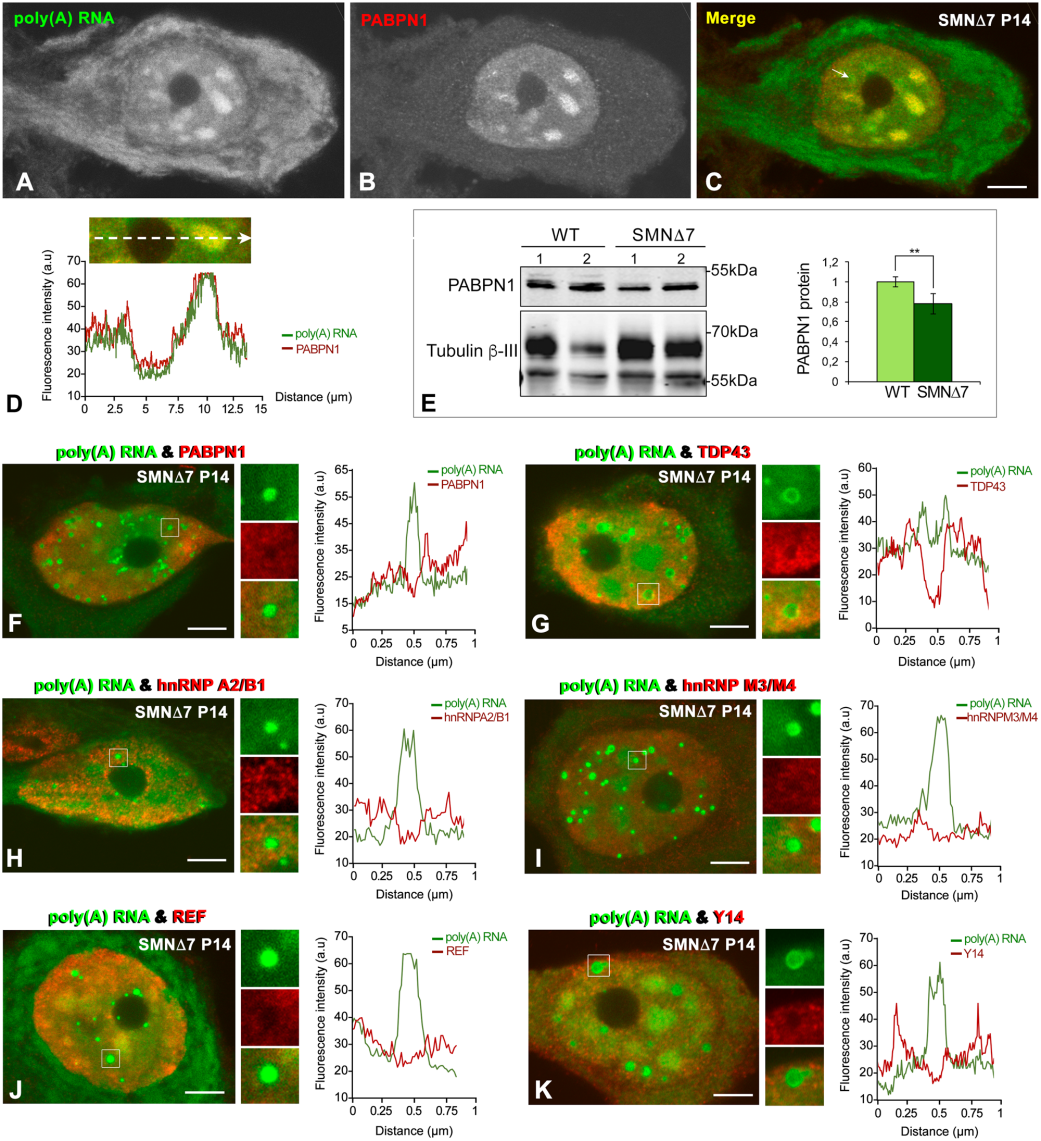
(**A**–**D**) Double labeling for poly(A) RNA and PABPN1 shows the colocalization of both molecules in nuclear speckles in a PARG-free MN from an SMN∆7 mouse. Scale bar: 5 µm. (**E**) Representative western blotting analysis of PABPN1 levels in spinal cord lysates from WT and SMN∆7 mice. Western blot bands for PABPN1 were normalized to Tubulin ß-III, which showed double immunoreactive bands at approximately 70 kDa and 55 kDa. We choose the 55 kDa band for normalization purposes since 50–55 kDa is the predicted and apparent molecular weight of Tubulin ß-III in WB analyses. The larger 70 kDa band observed could be due to cross-reactivity with a protein related to Tubulin ß-III or a post-translationally modified form of Tubulin ß-III. The bars represent a densitometric analysis of the WB bands for PABPN1 normalized to the 55 kDa Tubulin ß-III band and expressed as the mean ± SD of three independent experiments (WT (n = 3) vs SMN∆7 (n = 5)). *p* value from WT and SMN∆7 data comparison: 4.3E-3 (**p < 0.005). (**F**–**K**) Double labeling for poly(A) RNAs and the RNA-binding proteins PABPN1 (**F**), TDP43 (**G**), hnRNPA2/B1 (**H**), hnRNPM3/M4 (**I**), REF (**J**) and Y14 (**K**) reveals an absence of the colocalization of poly(A) RNA with these RNA-binding proteins in PARGs, which was confirmed by plots of their respective fluorescence intensity profiles across a line. Scale bar: 4 µm.

Since PABPN1 binds to the poly(A) tail of polyadenylated RNAs^4^ and is required for efficient poly(A) RNA export from the nucleus^49^, we investigated changes in its nuclear pattern and protein levels in WT and SMN∆7 mice. PABPN1 was concentrated in nuclear speckles in both WT and SMN∆7 MNs (Figs 6A, 7B–D), but not in PARGs (Fig. 7F), suggesting that PABPN1 is not linked to the poly(A) RNAs within these granules. Moreover, western blotting analysis revealed significantly reduced PABPN1 levels in the spinal cord of SMN∆7 mice compared with those of WT animals (Fig. 7E).

TDP43 plays a central role in the pathogenesis of MN diseases^50^. This protein is a component of the hnRNP particles that regulate the splicing of a variety of pre-mRNAs^50–52^. TDP43 immunolabeling showed a diffuse nuclear localization, excluding nuclear speckles and the nucleolus, in both WT and SMN∆7 MNs, but the protein was not detectable in pathological PARGs (Figs 6B, 7G). Similarly, immunostaining for two hnRNP family proteins, A2/B1 and M3/M4, which are involved in packing nascent pre-mRNA and in alternative splicing regulation^53,54^, revealed their diffuse nuclear distribution in both WT and SMN∆7 MNs (Figs 6C,D, 7H,I). No labeling of PARGs was detectable for the A2/B1 and M3/M4 proteins (Fig. 7H,I).

To further investigate the possible concentration of post-spliced mRNA-binding proteins in PARGs, we focused on the detection of REF/Aly and Y14, two RNA-binding proteins that are directly implicated in the nuclear trafficking and export of mRNAs from the nucleus^55,56^. REF and Y14 showed a diffuse nuclear localization, which excluded the nucleolus, in addition to being concentrated in nuclear speckles in both WT and SMN∆7 MNs (Figs 6E,F, 7J,K). Interestingly, neither export factor was accumulated in pathological PARGs (Fig. 7J,K). The lack of colocalization of REF/Aly and Y14 proteins with poly(A) RNA-positive PARGs was confirmed by the representation of their fluorescence intensity profiles across a line (Fig. 7F–K).

### Accumulation of intron-containing pre-mRNAs encoding essential proteins for MN function in the SMN∆7 mice

**Figure 8:**
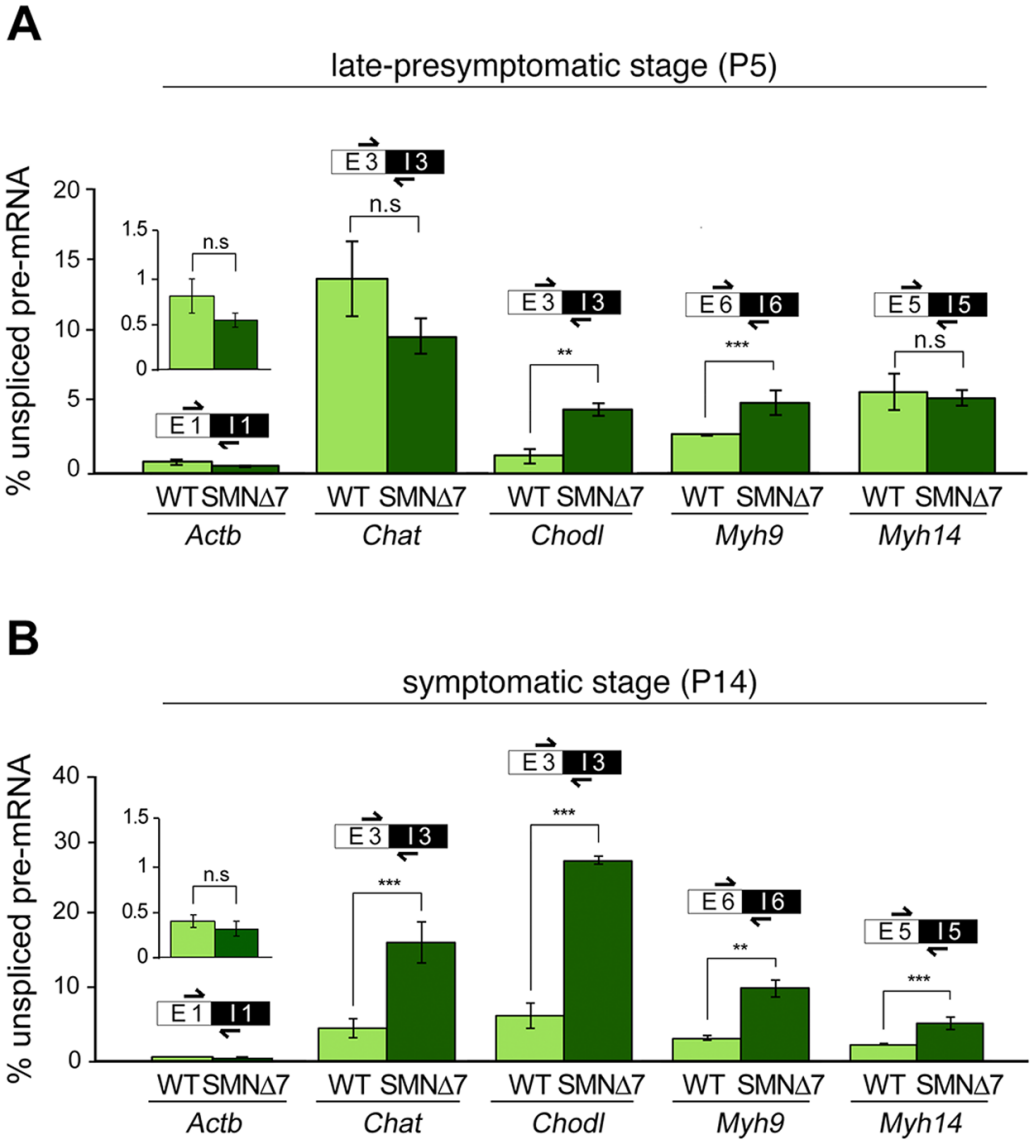
(**A**,**B**) qRT-PCR determination of the intron-containing (unspliced) *Chat, Chodl, Myh9* and *Myh14* pre*-*mRNAs reveals a significant increase of the relative abundance of their unspliced pre-mRNAs in the spinal cords of SMN∆7 mice compared with WT mice at P14 (**B**). *p* values from WT and SMN∆7 data comparison at P14: 2.1E-3 for *Chat*, 3,5E-4 for *Chodl*, 6.3E-4 for *Myh9* and 2.2E-4 for *Myh14* (**p < 0.005; ***p < 0.0005). (**A**) During the late presymptomatic stage (P5), this increase was only detected in *Chodl* and *Myh9* pre-mRNAs. *p* value from WT and SMN∆7 data comparison at P5: 2,1E-3 for *Chodl* and 3,5E-4 for *Myh9* (**p < 0.005; ***p < 0.0005). No significant changes (*n.s*) in unspliced *Actb* pre-mRNAs were detected when samples from WT and SMN∆7 mice were compared at either P5 or P14. The bars represent the mean ± SD of the relative fraction of the total RNA transcript that is unspliced pre-mRNA from WT (n = 3) and SMN∆7 mice (n = 5). qRT-PCR analyses were always confirmed in triplicate.

Previous studies have demonstrated that SMA severity correlates with the decreased assembly of spliceosomal snRNP complexes, which leads to widespread defects in the splicing of genes expressed in MNs^27,29,31^. On this basis, we investigated whether the nuclear accumulation of polyadenylated mRNAs in PARGs was associated with the splicing dysfunction of four genes, *Chat, Chodl, Myh9* and *Myh14*, which are important for MN maturation and synapse development and function^28,57^. These genes encode choline acetyltransferase (*Chat*) and chondrolectin (*Chodl*), which are processed by the major U2-dependent spliceosome, and the non-muscle myosin II isoforms IIA (*Myh9*) and IIC (*Myh14*), which are processed by the minor U12-dependent spliceosome. Splicing efficiency was analyzed by estimating the percentage of the unspliced (exon-intron sequence) forms of the *Chodl*, *Chat*, *Myh9* and *Myh14* pre-mRNAs by qRT-PCR in spinal cord RNA extracts. Importantly, we found that in relation to WT samples, samples from P5 SMNΔ7 animals had a significant increase in the proportion of unspliced forms of the *Chat* and *Myh14* mRNAs, while the accumulation of the unspliced forms was extended to the four examined pre-mRNAs (*Chodl*, *Chat*, *Myh9* and *Myh14)* during the late symptomatic stage (P14), (Fig. 8A,B). No significant changes in unspliced *Actb* (the beta-actin housekeeping gene) pre-mRNAs were detected when samples from WT and SMN∆7 mice were compared at P5 or P14 (Fig. 8A,B).

## Discussion

The present study demonstrates that the SMN deficiency in SMN∆7 MNs affects the nuclear distribution of polyadenylated mRNAs, resulting in their progressive accumulation in Sam68-positive PARGs. During the symptomatic stage, this neuronal response is associated with an increased proportion of intron-containing *Chat, Chodl, Myh9* and *Myh14* mRNAs. The accumulation of poly(A) RNAs in PARGs appears to be an early (P5) cellular manifestation of the nuclear mRNA metabolism dysfunction in SMA MNs, which could contribute to SMA pathogenesis. Moreover, MNs that contain numerous PARGs frequently exhibit other signs of neuronal dysfunction. These signs include the segregation of the granular component of the nucleolus and cytoplasmic reduction of polyadenylated mRNAs, which support defective ribosome biogenesis and translation, according to previous studies in SMA mice^33,34^. Collectively, the nuclear accumulation of poly(A) RNAs in PARGs is consistent with the splicing alterations reported here and in previous studies in SMA MNs^27–29,31^.

A poorly understood aspect of SMA is why MNs that carry the same deletion or mutation of the *SMN1* gene are affected differently. Our results confirm this heterogeneity, showing that at both P5 and P14, affected MNs carrying PARGs coexist with other MNs that have an apparently normal pattern of poly(A) RNA distribution. In this regard, differences in the pathogenic vulnerability of MNs have recently been correlated with individual differences in SMN protein levels in MNs from a particular SMA mouse^58^.

Although we have previously reported the presence of PARGs in sensory ganglion neurons upon proteasome inhibition^16,17^, the present work provides the first demonstration that these structures are present in SMA MNs. The PARG represents a distinct nuclear entity that is clearly distinguishable by its structure and molecular composition from other nuclear compartments, such as nuclear speckles^9^, Cajal bodies^43,59,60^, gems^44^ and clastosomes^45^. PARGs share with nuclear speckles the presence of poly(A) RNAs; however, PARGs lack spliceosomal snRNPs and display an ultrastructural configuration as sharply defined nuclear bodies that is clearly different from the interchromatin granule clusters of nuclear speckles^9,43^. Cajal bodies, the nuclear structures involved in spliceosomal snRNP biogenesis^42^, are depleted in SMN∆7 MNs^34^; additionally, the Cajal body marker coilin was not detected in PARGs. Similarly, gems, SMN-positive and coilin-negative nuclear bodies of unknown function^43,44^, have been reported in fetal MNs^61^, but they are absent in postnatal and mature MNs from both WT and SMN∆7 mice^34^. Moreover, we did not detect SMN in PARGs. Finally, these RNA granules also lack the 20S proteasome, a molecular marker of clastosomes, which are nuclear proteolytic factories enriched in catalytic proteasomes, ubiquitylated proteins and proteasome substrates^45,62^.

PARG-containing MNs were already observed at P5, but they increased in number during the late symptomatic stages (P14), presumably reflecting the well-established asynchrony at the beginning of the MN degeneration in SMN∆7 mice^63^. We propose that the formation of PARGs in SMN∆7 MNs reflects a stress-related dysfunction of RNA metabolism, essentially in pre-mRNA splicing. Consistent with this view, pre-mRNA splicing has emerged as a well-known and important target of several stressing agents, resulting in alternative splicing dysregulation and splicing inhibition^64^. In the case of SMA, several studies demonstrated that the hyperactivation of the endoplasmic reticulum stress pathway and widespread defects in splicing underlie the neurodegeneration observed in SMA MNs^27,29,31,32,65^. Moreover, we have previously reported the formation of PARGs following proteasome inhibition-induced proteotoxic stress in rat sensory ganglion neurons, an experimental condition that produces a dysfunction of RNA metabolism and a disruption of the protein synthesis machinery^16,17^.

An important finding in this study is the accumulation of Sam68 in PARGs. Sam68 is a member of the STAR (signal transducer and activator of RNA) family of RNA-binding proteins that bind both RNA and DNA and are involved in signal transduction, transcription and alternative splicing regulation^36,66–68^. As a splicing regulator, Sam68 may promote exon inclusion or exclusion in certain neural pre-mRNAs, including *SMN2*, *Bcl-x* and *Nrxn 1*^36,37,47^. Regarding *SMN* genes, Sam68 is a physiological regulator of *SMN2*, but not of *SMN1*, splicing. Thus, Sam68 directly binds to the *SMN2* pre-mRNA and acts as a splicing repressor of exon 7 inclusion in *SMN2* transcripts^46,69^. In the present study, we detected no significant changes in the mRNA and protein levels of Sam68 in the spinal cords of SMN∆7 mice compared with the WT animals. Although the accumulation of Sam68 in PARGs should reduce its nucleoplasmic levels, potentially facilitating the inclusion of exon 7 in *SMN2* transcripts^46,69^, its partial relocation in PARGs does not correct the defective *SMN2* splicing in SMN∆7 mice. In fact, SMN protein levels are dramatically reduced in the spinal cords of the SMN∆7 mice during the symptomatic stages (present results and^34^). However, our results show changes in the alternative splicing regulation of the Sam68-dependent *Bcl-x* and *Nrxn-1* mRNAs in the spinal cords of SMN∆7 mice. It is well-established that Sam68 binds *Bcl-x* mRNA, and its intracellular levels regulate the balance of alternative splicing to produce pro-apoptotic *Bcl-x(s)* transcripts or anti-apoptotic *Bcl-x(L)* transcripts^48^. In particular, whereas the upregulation of Sam68 enhances *Bcl-x(s)* splicing, its downregulation promotes the production of *Bcl-x(L)* transcripts^47^. In this context, our finding of a reduction of the *Bcl-x(s)/Bcl-x(L)* ratio in the SMN∆7 mice, compared with WT littermates, is consistent with a downregulation of Sam68.

Regarding *Nrxn1* transcripts, changes in alternative splicing regulation result in a decreased *Nrxn1 4(*−*)/Nrxn1 4*(+) ratio, with a relative increase in the isoform that includes exon 20 in *Nrxn1* alternatively spliced segment 4 (AS4). Importantly, the neurexin protein 4(+) and 4 (−) variants show different interactomes with proteins that are key mediators of synaptic formation and maintenance, including neuroligins (for a review, see^37^). Thus, previous studies have demonstrated that neurexin 4(+) exhibits weak binding with the neuroligin-1B postsynaptic receptor, which affects synaptic adhesion^70,71^. In this context, the Sam68-dependent relative abundance of neurexin 4(+) in SMN∆7 mice might contribute to the synaptic dysfunction observed in SMA MNs^22,25^. Collectively, our results suggest that the recruitment of Sam68 into PARGs impacts on its normal function in splicing regulation. In this line, recent studies have shown that Sam-68 deficient preadipocytes exhibit alternative splicing imbalances in components of the mTOR signaling pathway that lead to defective adipogenic differentiation^72^.

The nuclear accumulation of immature or aberrant pre-mRNAs can be triggered by a wide range of errors in mRNA processing, which prevent the recruitment of mRNA export factors^12^. We believe that PARGs may contain incorrectly processed polyadenylated transcripts bound to Sam68, but lack other RNA-binding proteins involved in packing nascent mRNA (hnRNPA2/B1), splicing regulation (hnRNPM3/M4 and TDP43) and polyadenylation (PABPN1). Consistent with the nuclear accumulation of poly(A) RNA in PARGs, REF and Y14, two RNA-binding proteins that link pre-mRNA splicing to nuclear export^73,74^, are not detectable in these granules. Moreover, we observed a reduction of the levels of PABPN1, a protein that, in addition to polyadenylation, is also involved in mRNA export from the nucleus^49^. Therefore, a deficiency of this export factor in SMN∆7 MNs could also contribute to the accumulation of poly(A) RNA in PARGs.

Alternative splicing defects account for nearly 50% of human inherited diseases^75^, and missplicing events are particularly prominent in neurodegenerative diseases^76,77^. Previous studies in mouse models of SMA have demonstrated SMN-dependent downregulated spliceosomal snRNPs and altered alternative splicing, preferentially in U12-dependent introns^27–29,31^. Our results extend the splicing defects in SMA MNs. Thus, we demonstrate here the accumulation of incompletely spliced (intron-containing) *Chat, Chodl, Myh9* and *Myh14* pre-mRNAs during the late symptomatic stage (P14), when a higher incidence of PARG-containing MNs occurs. Interestingly, the accumulation of intron-containing mRNAs impacts the transcripts processed by both the minor (*Myh9* and *Myh14)* and major (*Chat, Chodl)* spliceosomes, suggesting a global splicing dysfunction. Importantly, reduced *Chodl* expression has been demonstrated in SMA mouse MNs and linked to MN outgrowth defects^28,78^.

Although MN functions, such as axonal RNA transport and translation control at the neuromuscular junction, are altered in SMA^79^, spliceosome dysfunction plays a critical role in SMA pathogenesis (for a review, see^30^). We propose that the abnormal accumulation of polyadenylated RNAs in PARGs reported here, together with the previously demonstrated depletion of canonical (coilin-, SMN- and snRNP-positive) Cajal bodies^34,80,81^ are two cellular manifestations of the global splicing dysfunction in SMA MNs. Notably, an enhancement of the nuclear poly(A) RNA signal, which is associated with the formation of cytoplasmic stress granules, has been reported in a cellular model of *C9orf*7*2* amyotrophic lateral sclerosis^82^, suggesting that the nuclear accumulation of polyadenylated mRNAs may be a more general mechanism in the pathogenesis of MN diseases.

The reduction of the cytoplasmic poly(A) RNA signal in MNs containing a large number of PARGs supports a decrease in the translation efficiency, with reduced availability of polyadenylated mRNAs for protein synthesis. Consistent with this notion, by ultrastructural and immunocytochemical analyses, we have previously reported nucleolar alterations and a severe disruption of the protein synthesis machinery in MNs from the SMN∆7 mice^34^. Furthermore, a recent study on the transcriptome and translatome in SMA mice, by means of next-generation sequencing, provides an important catalogue of the mRNAs with altered translation efficiency^33^. The negative impact of the reduced levels of cytoplasmic poly(A) RNAs on translation might be reinforced by the failure of SMN∆7 MNs to recruit these RNAs into cytoplasmic stress granules, which is a protective cellular mechanism for the transient storage and stabilization of mRNAs during the stress-induced inhibition of translation^40^.

In conclusion, SMN deficiency in SMN∆7 MNs causes an abnormal nuclear accumulation of polyadenylated RNAs in PARGs and the cytoplasmic depletion of these RNAs. This neuronal response suggests a defective mRNA processing, export and translation and is also consistent with the widespread splicing defects reported in SMA MNs. The present study provides additional support for the hypothesis that the dysfunction of nuclear mRNA metabolism plays a critical role in MN degeneration and consequently in SMA pathogenesis.

## Material and Methods

### Animals

The *Smn*^+/−^*, SMN2*^+/+^*, SMN****∆***7^+/+^, heterozygous knockouts for mouse *Smn* (FVB.Cg-Tg[SMN2*delta7]4299Ahmb Tg[SMN2]89Ahmb Smn1tm1Msd/J, stock number 005025), which were purchased from The Jackson Laboratory (Sacramento, USA), were crossed to generate *Smn*^−/−^*, SMN2*^+/+^*, SMN***∆***7*^+/+^ (hereafter referred to as SMN∆7) mice and *Smn*^+/+^*, SMN2*^+/+^*, SMN***∆***7*^+/+^ mice that were wild-type for *Smn* (hereafter referred to as WT). SMN∆7 mice exhibit a severe postnatal SMA phenotype with a mean lifespan of approximately two weeks^63,83^. Animal care and handling were performed in accordance with the Spanish legislation (Spanish Royal Decree 53/2013 BOE) and the guidelines of the European Commission for the Accommodation and Care of Laboratory Animals (revised in Appendix A of the Council Directive 2010/63/UE). The experimental plan was examined and approved by the Ethics Committee of the University of Cantabria and the Committee for Animal Care and Use of the University of Lleida. On postnatal day (P) 0, the identification of WT and SMN∆7 mice was carried out by genotyping with PCR. DNA was extracted from the tail, as previously described^63^. Age-matched WT littermates of mutant animals were used as controls.

### Immunofluorescence and confocal microscopy

For immunofluorescence, four mice per group (WT and SMN∆7) at P5 and P14 were perfused, under deep anesthesia with pentobarbital (50 mg/kg), with 3.7% paraformaldehyde (freshly prepared) in phosphate-buffered saline (PBS). The spinal cords were rapidly dissected, removed, post-fixed for 6 hours and washed in PBS. Transverse sections (160 µm thick) of the spinal cord were obtained with a vibratome, and small tissue fragments from the anterior horn were dissected out. The samples were transferred to a drop of PBS on a positively charged slide (Superfrost Plus, Thermo Scientific, Germany), and squash preparations of dissociated MNs were generated following the previously reported procedure^84^. The samples were sequentially treated with 0.5% Triton X-100 in PBS for 45 minutes, 0.1 M glycine in PBS containing 1% bovine serum albumin (BSA) for 30 minutes and 0.05% Tween 20 in PBS for 5 minutes. Then, the samples were incubated for 3 hours with the primary antibody containing 1% BSA at room temperature, washed with 0.05% Tween 20 in PBS, incubated for 45 minutes in the specific secondary antibody conjugated to FITC, TexasRed or Cy3, or Cy5 (Jackson, USA) and counterstained with PI for the detection of nucleic acids. The slides were then washed in PBS and mounted with the ProLong Anti-Fading Medium (Invitrogen) or Vectashield (Vector, USA).

Confocal images were obtained with an LSM510 (Zeiss, Germany) laser scanning microscope using a 63x oil (1.4 NA) objective. To avoid overlapping signals, images were obtained by sequential excitation at 488 nm, 543 nm and 633 nm, to detect FITC, TexasRed or Cy3, and Cy5, respectively. Fluorescence profiles of confocal intensity signals across a line were generated to analyze the spatial association between PARGs and the nuclear speckles (TMG-Cap), Cajal bodies (coilin), gems (SMN), clastosomes (20S proteasome) and the RNA-binding proteins PABPN1, Sam68, TDP43, hnRNP A2/B1, hnRNP M3/M4, REF and Y14. Images were processed using Photoshop software.

The following primary antibodies were used for immunofluorescence: the mouse monoclonal antibodies anti-SMN (610646, BD Transduction Laboratories, USA), anti-TMG-cap (NA02A, Oncogene), anti-REF (ab6141, Abcam), anti-Y14 (ab5828, Abcam) and anti-hnRNPM3/M4 (ab9548, Abcam), the rabbit polyclonal antibodies anti-PABPN1 (sc-67017, Santa Cruz Biotechnology), anti-proteasome 20 S subunit α5 (ab11437, Abcam), anti-Sam68 (sc-333, Santa Cruz Biotechnology), anti-coilin 204/10 (204.3 serum^85^), and anti-TDP43 (10782-2-AP, Proteintech Inc. Group), and the goat polyclonal antibody anti-hnRNPA2/B1 (sc-10035, Santa Cruz Biotechnology).

### *In situ* hybridization and quantification

Tissue fragments from the ventral horn of the spinal cord fixed with 3.7% paraformaldehyde were processed for fluorescence *in situ* hybridization (FISH). MN perikarya were then dissociated as described above. Preparations of MNs were permeabilized with TBS-E-SDS for 15 minutes at 37 °C, washed three times in 6x SSPE-0.1% Tween 20 for 15 minutes, and incubated with the probe containing tRNA for 3 hours at 42 °C in a humidified chamber. An oligo dT _(50)-_mer, 5′-end labeled with biotin (MWG-Biotech, Germany) was used as a probe for fluorescence *in situ* hybridization (FISH) to poly(A) RNA. The hybridization mixture contained 80 ng of oligo dT(50), 2xSSC, 1 mg/ml tRNA, 10% dextran sulfate and 25% formamide. After hybridization, the MNs were washed in 6xSSC for 15 minutes, and then washed in 4x SSC-0.1% Tween 20 for 15 minutes at room temperature. The hybridization signal was detected with FITC-avidin for 30 minutes. For the amplification of the hybridization signal, neuronal samples were incubated with avidin-biotin for 30 minutes, washed in 4x SSC-0.1% Tween 20 for 15 minutes and then incubated with FITC-avidin for 30 minutes. All samples were mounted with Vectashield (Vector, USA). Some samples were also processed for double- or triple-labeling experiments combining poly(A) RNA detection with immunofluorescence for TMG-cap, PABPN1, Sam68, TDP43, hnRNP A2/B1, hnRNPM3/M4, REF or Y14.

The quantitative analysis of the proportion of SMN∆7 MNs containing PARGs was performed in squash preparations processed for FISH with the poly(dT) probe. The proportion of neurons containing these granules was estimated by direct examination of the different focal planes throughout neuronal nuclei, using a 40 × objective. Quantification was performed on at least 100 MNs from three WT and SMN∆7 mice.

The fluorescence intensity of poly(A) RNAs in the Nissl substance was quantified using the confocal Zeiss LSM 5 image analysis system. At least 30 confocal images of MNs per animal from WT (n = 3) and SMN∆7 (n = 3) mice were recorded using a 63x oil objective. For each MN, four measurements of poly(A) RNA signal intensity in the peripheral cytoplasm, where the RNA-rich Nissl substance is largely distributed, were made. The values were corrected for background staining by subtraction of a blank measurement taken outside the cell.

### Electron microscopy

For conventional ultrastructural examination of MNs, WT and SMN∆7 mice (n = 3 per group) were perfused under deep anesthesia with 3% glutaraldehyde in 0.1 M phosphate buffer, pH 7.4. Transverse sections (500 µm thick) were obtained with a vibratome, and anterior horn tissue fragments were dissected out, rinsed in 0.1 M phosphate buffer, postfixed in 2% osmium tetroxide, dehydrated in acetone and embedded in araldite (Durcupan, Fluka, Switzerland). Ultrathin sections stained with uranyl acetate and lead citrate were examined with a JEOL 201 electron microscope.

For immunoelectron microscopy of Sam68, WT and SMN∆7 mice were perfused with 3.7% paraformaldehyde in 0.1 M phosphate buffer. Tissue fragments of the ventral horn were washed in 0.1 M cacodylate buffer, dehydrated in increasing concentrations of methanol at −20 °C, embedded in Lowicryl K4M at −20 °C and polymerized with ultraviolet irradiation. Ultrathin sections were mounted on nickel grids and sequentially incubated with 0.1 M glycine in PBS for 15 min, 5% BSA in PBS for 30 min and the primary antibody for 2 h at 37 °C. After washing, the sections were incubated with the specific secondary antibodies coupled to 10 nm gold particles (BioCell, UK; diluted 1:50 in PBS containing 1% BSA). Following immunogold labeling, the grids were stained with lead citrate and uranyl acetate and examined with a JEOL 201 electron microscope. As controls, ultrathin sections were treated as described above without the primary antibody.

### Real time quantitative PCR (qRT-PCR) for relative gene expression analysis

Five SMNΔ7 (n = 5) and 3 WT (n = 3) mice were used for qRT-PCR studies. The mice were decapitated after being anesthetized and the lumbar spinal cord was quickly removed and frozen in liquid nitrogen. RNA was isolated with Trizol following the manufacturer’s instructions (Invitrogen, Carlsbad) and purified with the RNeasy kit (Qiagen, Hilden, Germany).

One microgram of RNA was reverse-transcribed to first-strand cDNA using a High Capacity cDNA Reverse Transcription Kit (Life Technologies) using random hexamers as primers. The cDNA concentration was measured in a spectrophotometer (Nanodrop Technologies ND-1000) and adjusted to 0.5 μg/μl. The expression of the mRNAs candidates *Sam68* (Sam68), *Actb* (ß-actin), *Chat* (choline O-acetyltransferase), *Chodl* (chondrolectin), *Myh9* (Myosin-9) and *Myh14* (Myosin-14), as well as the long and short isoforms of *Bcl-x* (Bcl-XL and Bcl-XS), and *Nrxn1 4*(−) *and Nrxn1 4*(+) variants (neurexin 4(+) and neurexin 4(−)) was determined by qRT-PCR using gene-specific SYBR Green- based primers (Takara). RT-qPCRs was performed with three biological replicates and technical triplicates/duplicates of each cDNA sample. The threshold cycle (Ct) for each well was determined. The results were normalized to the *45 S* ribosomal gene transcripts, which had no significant variation in expression across the sample sets and was therefore chosen as a normalizer in our experiments. Relative gene expression was calculated according to the 2−(ΔΔCt) equation^86^. Each value in this work represents the mean ± SD of independent samples obtained under the same conditions and compared to two replicated qRT-PCR analyses. The SYBR Green-based specific primers for murine RNAs were as follows: for the *Sam68* mRNA 5′-CTCCAGCTAGGCCAGTGAA-3′ and 5′-TTGTGGGTAAAGCAACAGGA-3′; for the unspliced pre-mRNA of *Actb* containing intron 1, 5′-GACTCCCAGCAC ACTGAACT-3′ and 5′-CTCAGGGCAGGTGAA ACTGT-3; for the spliced mRNA of *Actb* 5′-CAGCCTTCCTTCTTGGGTATG-3′ and 5′-GGCATAGAGGTCTTT ACGGATG-3′; for the unspliced pre-mRNA of *Chat* containing intron 3, 5′-CTTGGGGCCAGTCTGATAGC-3′ and 5′-GGACACATGGCTAGAAGGGG-3′; for the spliced mRNA of *Chat*, 5′-CTGGCTTACTACAGGCTTTACC-3′ and 5′-GTGGCCGATCTGATGTTGT-3′; for the unspliced pre-mRNA of *Chodl* containing intron 3, 5′-GCTGTTGTCTCCCGCATCTT-3′ and 5′-AAGTGGAAGCGTTTGGGA TT-3′; for the spliced mRNA of *Chodl*, 5′-GAAGCAGGCATAATTCCCAATC-3′ and 5′-TTCCCAAAGCA ACCAGTATCA-3; for the unspliced pre-mRNA of *Myh9* containing intron 6, 5′-CACTTGTGTGGCATTGGGAC-3′ and 5′-AAGAAGGACCT CTCCTCCGA-3′; for the spliced mRNA of *Myh9*, 5′-TGGTGCCAACATTGA GACTTAT-3′ and 5′-CCAGACAGCAG GTAGTAGAAGA-3′; for the unspliced pre-mRNA of *Myh14* containing intron 5, 5′-TCACCCTGAAAAGATGCCCC-3′ and 5′-TCGAAGGTCCAGACAGGCAT-3′; for the spliced mRNA of *Myh14*, 5′-GGAGCA AACATCGAGACCTATC-3′ and 5′-GGTAGAAGATATGGAAGCTGCATT-3′. For the long form of *Bcl-x (Bcl-x(L))*, 5′-GAGTTACCGGCGACCCA-3′ and 5′-CGACAGCAA GCAGGTACAA-3′; for the short form of *Bcl-x (Bcl-x(s))*, 5′-GCTGCCTACCAGAACCTTATC-3′and 5′-GGCTCAACCAGTCCATTGT-3′; for the exon 20 containing *Nrxn1* variant (*Nrxn1 4*(+)), 5′-TAGTTGATGAATGGCTACTCGACAAA-3′ and 5′-GACTCAGTTG TCATAGAGGAAGGCAC-3′; for the exon 20 skipped *Nrxn1* variant (*Nrxn1 4*(−)), 5′-GCTACCCTGCAGGGCGT-3′ and 5′-GAGGTGGACATCTCAGACTGCAT-3′.

### SDS-PAGE and immunoblotting

Spinal cords from WT (n = 3) and SMNΔ7 mice (n = 5) were lysed at 4 °C in a buffer containing 50 mM Tris (pH 8), 150 mM NaCl, 2% Nonidet P-40, 1 mM MgCl_2_, 1 mM dithiothreitol, and 10% glycerol, and supplemented with EDTA-free complete protease inhibitor cocktail and PhosSTOP (Roche). The spinal cord samples were sonicated for 5 cycles of 30 seconds ON/OFF at 4 °C using a Bioruptor Plus (Diadode) and left on ice for 20 min. The samples were then cleared by centrifugation at 14,000 rpm for 10 min at 4 °C. The proteins were separated on 4–20% NuPage TG SDS–PAGE gels (Invitrogen) and transferred to nitrocellulose membranes using standard procedures. Mouse monoclonal anti-Tubulin ß-III (Sigma T8660) and rabbit polyclonal anti-PABPN1 and anti-Sam68 were used. Protein bands were detected with an Odyssey^TM^ Infrared-Imaging System (Li-Cor Biosciences) according to the Odyssey^TM^ Western-Blotting Protocol. Immunoblots were developed with anti-mouse IRDye800DX or anti-rabbit IRDye680DX (Rockland Immunochemicals, USA) secondary antibodies. For the quantitative analysis of the blots, ImageJ software was used (U. S. National Institutes of Health, Bethesda, Maryland, USA, http://imagej.nih.gov/ij).

### Statistical analysis

For comparisons between WT and SMN∆7 samples, data were analyzed using the GraphPad Prism 7 software and an unpaired Student’s *t*-test. Significance was established at *p* ≤ 0.05.

## Electronic supplementary material

Supplementary Figures
