# Supplementary material for: Accumulation of poly(A) RNA in nuclear granules enriched in Sam68 in motor neurons from the SMNΔ7 mouse model of SMA
Source: Sci Rep. 2018 Jun 25;8:9646. doi: 10.1038/s41598-018-27821-3 (PMC6018117; doi:10.1038/s41598-018-27821-3)
Supplement: Supplementary file 1 — Supplementary Figures [file 41598_2018_27821_MOESM1_ESM.pdf]

Accumulation of poly(A) RNA in nuclear granules enriched in Sam68 in motor neurons from the SMNΔ7 mouse model of SMA

J. Oriol Narcís[1*], Olga Tapia[1*], Olga Tarabal[2], Lídia Piedrafita[2], Jordi Calderó[2], Maria T. Berciano[1,3] and Miguel Lafarga[1]

1. Department of Anatomy and Cell Biology and "Centro de Investigación Biomédica en Red sobre Enfermedades Neurodegenerativas (CIBERNED)", University of Cantabria-IDIVAL, Santander, Spain.

2. Department of Experimental Medicine, School of Medicine, University of Lleida and "Institut de Recerca Biomèdica de Lleida" (IRBLLEIDA), Lleida, Spain.

3. Present address: Department of Molecular Biology and CIBERNED, University of Cantabria-IDIVAL, Santander, Spain.

* These authors contributed equally to the work reported here

Abbreviated Title: Nuclear retention of poly(A) RNA in SMA motor neurons

Corresponding author:
Dr. Miguel Lafarga
Department of Anatomy and Cell Biology
Faculty of Medicine
Avd. Cardenal Herrera Oria s/n
39011 Santander
Spain
Fax: 34 942 201903
e-mail: lafargam@unican.es

Supplemental Information

Supplemental Figures

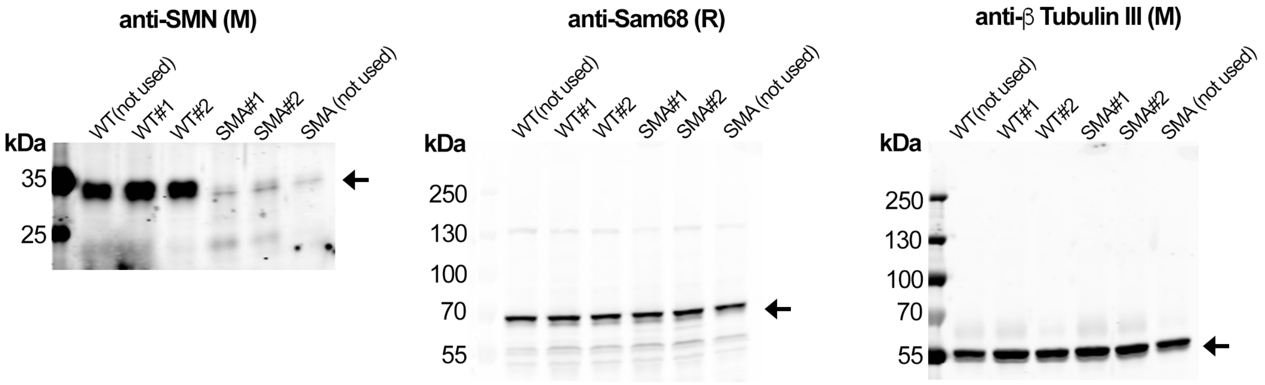

Figure S1. Uncropped blots probed with SMN, Sam68 and β–Tubulin III

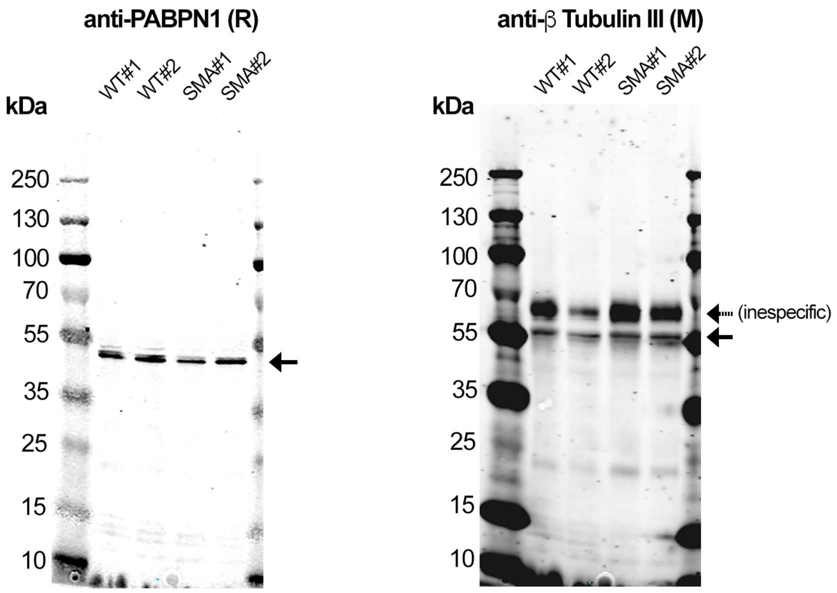

Figure S2. Uncropped blots probed with PABPN1 and β–Tubulin III
